# Evidence of a cognitive bias in the quantification of COVID-19 with CT: an artificial intelligence randomised clinical trial

**DOI:** 10.1038/s41598-023-31910-3

**Published:** 2023-03-25

**Authors:** Bogdan A. Bercean, Andreea Birhala, Paula G. Ardelean, Ioana Barbulescu, Marius M. Benta, Cristina D. Rasadean, Dan Costachescu, Cristian Avramescu, Andrei Tenescu, Stefan Iarca, Alexandru S. Buburuzan, Marius Marcu, Florin Birsasteanu

**Affiliations:** 1 Rayscape, 5, Nicolae Iorga, 010431 Bucharest, Romania; 2 Department of Radiology, Pius Brinzeu County Emergency Hospital, 156, Liviu Rebreanu, 300723 Timisoara, Romania; 3 Politehnica University of Timișoara, 2, Victoriei Square, 300006 Timisoara, Romania; 4 Victor Babeş University of Medicine and Pharmacy, 2, Eftimie Murgu Square, 300041 Timisoara, Romania; 5 The University of Manchester, Oxford Rd, Manchester, M13 9PL UK

**Keywords:** Computed tomography, Randomized controlled trials

## Abstract

Chest computed tomography (CT) has played a valuable, distinct role in the screening, diagnosis, and follow-up of COVID-19 patients. The quantification of COVID-19 pneumonia on CT has proven to be an important predictor of the treatment course and outcome of the patient although it remains heavily reliant on the radiologist's subjective perceptions. Here, we show that with the adoption of CT for COVID-19 management, a new type of psychophysical bias has emerged in radiology. A preliminary survey of 40 radiologists and a retrospective analysis of CT data from 109 patients from two hospitals revealed that radiologists overestimated the percentage of lung involvement by 10.23 ± 4.65% and 15.8 ± 6.6%, respectively. In the subsequent randomised controlled trial, artificial intelligence (AI) decision support reduced the absolute overestimation error (*P* < 0.001) from 9.5% ± 6.6 (No-AI analysis arm, n = 38) to 1.0% ± 5.2 (AI analysis arm, n = 38). These results indicate a human perception bias in radiology that has clinically meaningful effects on the quantitative analysis of COVID-19 on CT. The objectivity of AI was shown to be a valuable complement in mitigating the radiologist’s subjectivity, reducing the overestimation tenfold.

Trial registration: https://Clinicaltrial.gov. Identifier: NCT05282056, Date of registration: 01/02/2022.

## Introduction

The COVID-19 pandemic has created new ways in which existing and developing technologies are used in radiology. Although definitive diagnosis relies on real-time reverse-transcriptase-polymerase chain reaction (RT–PCR), CT still plays an essential role in the screening and monitoring of COVID-19 evolution, setting patient discharge criteria^1–4^, and is a valuable modality for measuring the extent of lung involvement.

**Figure 1:**
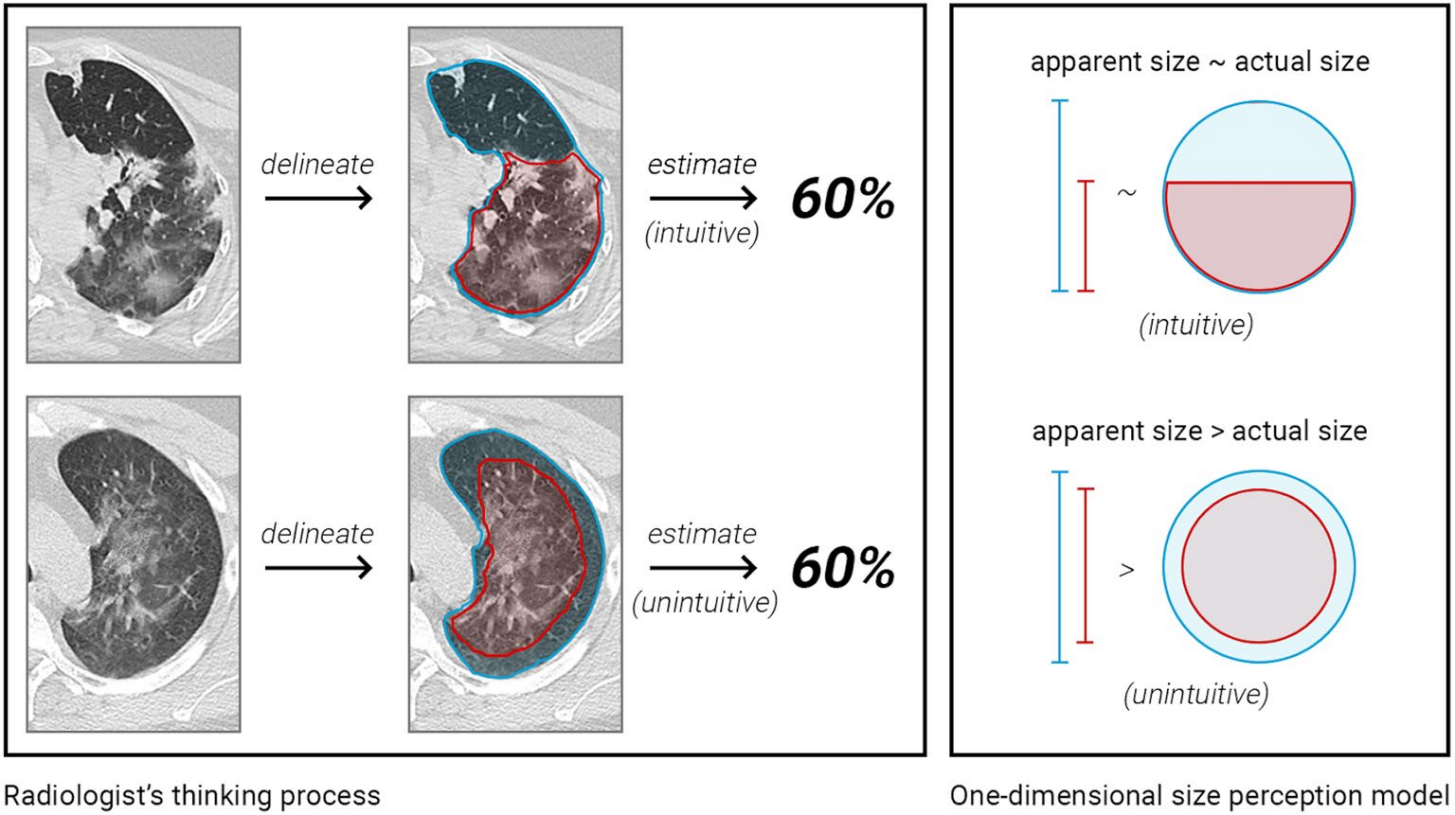
COVID-19 quantification judgement process (left) compared to the circle size comparison problem (right). In both cases, the apparent proportion of COVID-19 involvement (red) in relation to total lung area (blue) appears greater in the bottom examples. This is due to the overinfluence of a primary linear dimension (1D segment ratio = 80%) that is often insufficiently adjusted to the 2D context (2D circle ratio = 60%). This leads to an overestimation of the perceived percentages.

Radiologists measure pulmonary involvement in COVID-19 using either a quantitative assessment of the overall involvement^2^ or semiquantitative severity scores at the lobe level^4–6^.These markers were shown to be correlated with clinical outcomes and are often a key part of the treatment course^7^. Regardless of the scoring type, the assessment of lung involvement is a two-step thinking process: the radiologist first identifies the affected areas and then estimates a percentage of lung damage (Fig. 1).

Area judgement is a century-old field of study in cognitive psychology, as human beings exhibit an acute lack of precision in visual geometric comparisons^8,9^. Multiple authors have suggested a distinction between “apparent size” and actual “physical size”^10,11^. Although there is sometimes a 1:1 relation, in circle comparisons, for example, the apparent size is often larger than the physical size^10^. Krider et al.^11^ explained this by arguing that the human brain makes an initial comparison of two figures based on a single, linear dimension (e.g., height) that is most salient to the brain. The brain then completes the rest of the comparison by insufficiently adjusting the second dimension (e.g., width). Therefore, the effect is present on 2-dimensional (2D) comparisons and further amplified in 3 dimensions (3D), as is the case for CT. Yet, so far, only various other types of cognitive challenges across radiology have been studied^12^. The Fleischner Society^13^ briefly warned of the cognition-related risks involved in lung nodule growth estimation. More recently, Patel et al.^14^ showed evidence of an anchoring cognitive bias during the pandemic concerning the coexistence of other misdiagnosed respiratory syndromes. There is still a clear gap between the well-studied, comprehensive acknowledgement of general human cognitive biases^15^ and the narrower little studied effects of cognition problems in radiology.

Despite the wide adoption of lung involvement scores, the area judgement cognitive bias remains unaddressed in radiology. For the first time, radiologists are required to geometrically compare such irregular and disparate shapes, on such a large scale and all the available methods have one common denominator: the reliance on the reader's volume perception. This study analyses the perception risks of these measures and clinically tests an AI-based mitigation strategy.

To statistically confirm if this problem exists, we first conducted two experiments that analyse the two-step thinking process of a radiologist analysing CTs for COVID-19. The first experiment isolated and investigated the estimation step in simulated data, then the second examined the bias’s effect over the whole process. The primary hypothesis (H1) is that this geometric ratio assessment is prone to an overestimation bias. Next, a randomised clinical trial was conducted to study whether the bias could be mitigated by using a commercial AI clinical support system. The secondary hypothesis (H2) is that the reader’s objectiveness can be improved with the use of computer-aided diagnosis (CAD). As the study does not propose to address the development process of a new AI CAD system, it employs Rayscape^16^, an existing commercial medical device.

## Results

### Bias validation in the synthetic experiment

**Figure 2:**
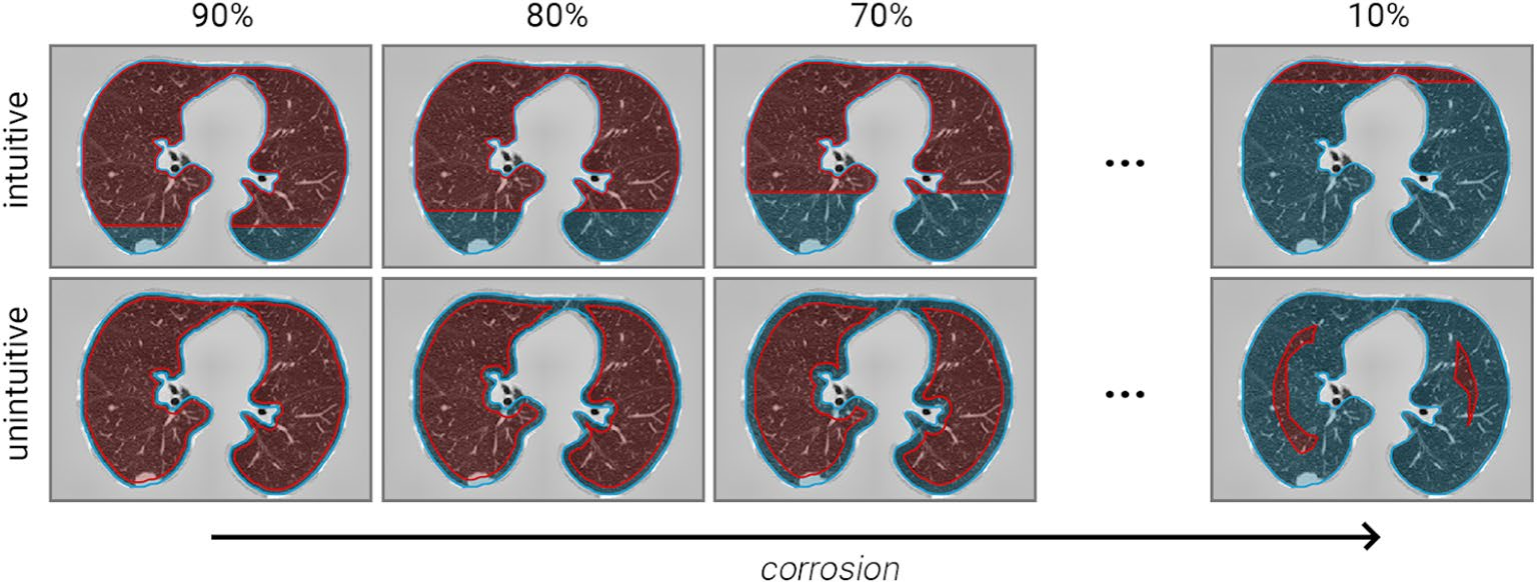
Synthetically crafted lung CT slices. The same starting slice was predelineated differently to simulate real involvement rates (18 slices and 9 involvement rates in total). Samples shown on the bottom row (unintuitive) should be more susceptible to overestimation, according to area perception theory.

The first experiment involved 40 radiologists who answered a survey regarding 18, nine intuitive and nine unintuitive, synthetically generated images (Fig. 2), inspired by the general area perception model in Fig. 1. Then, each radiologist estimated the level of pulmonary involvement (red) as the percentage of the total lung area (red and blue) that was affected.

**Figure 3:**
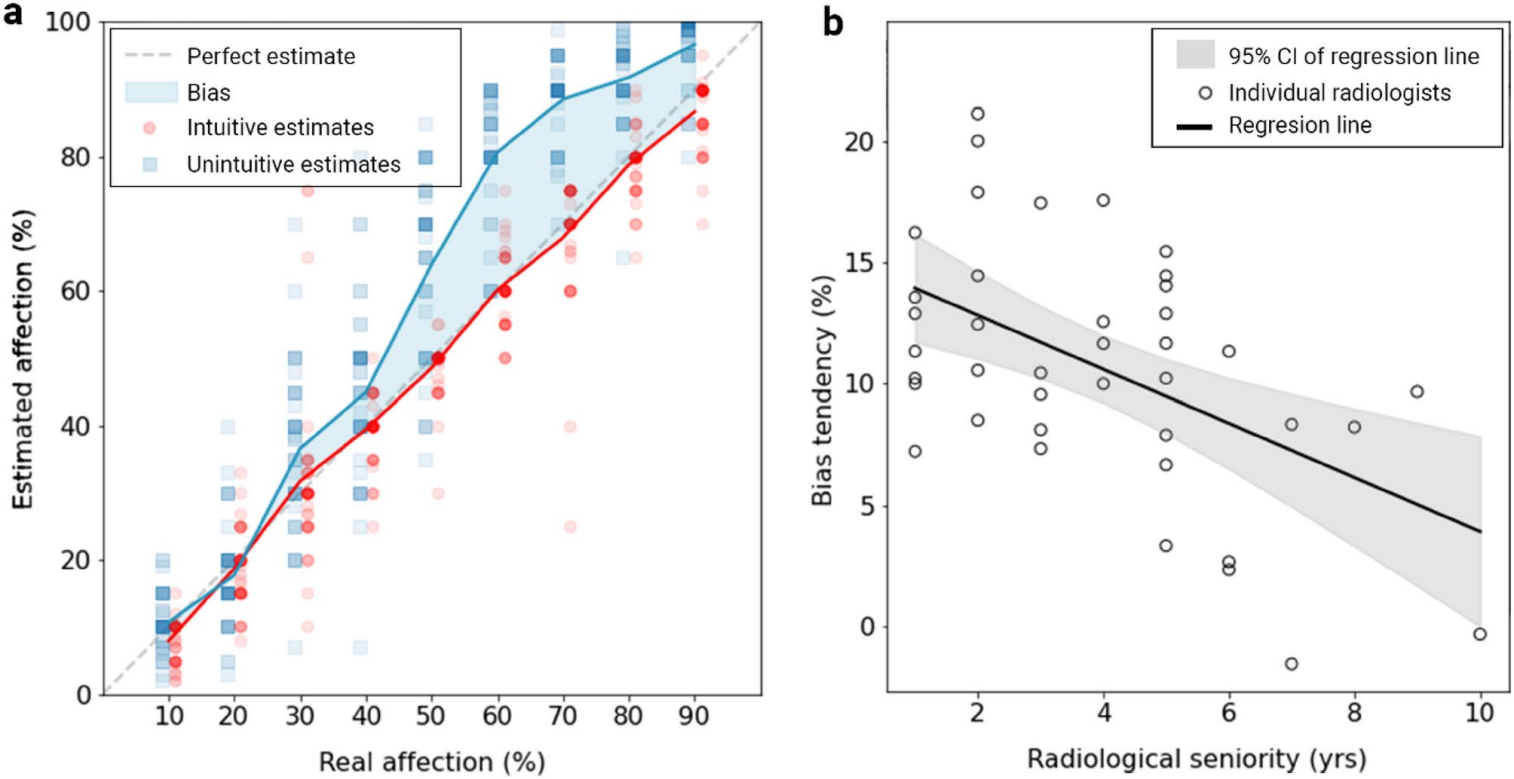
COVID-19 involvement overestimation across synthetic cases and radiological seniority. (**a**) Estimated involvement distribution for 18 synthetic cases. The estimates tend to diverge for the higher involvement percentages. (**b**) The bias tendency (mean difference in the overestimation) as a function of radiological seniority, using simple linear regression.

We compared the radiologists’ mean errors over the nine intuitive cases against the mean errors over the nine unintuitive (bias-susceptible) cases. We found that radiologists, on average, could be objective in judging the geometric ratios in the basic samples (mean difference = 1.193%), but they showed an overestimation bias in the unintuitive cases (*t*(39) = 12.885%, *P* < 0.001), with a mean overestimation difference of 10.280 ± 4.540% (95% CI). When grouped by involvement severity (Fig. 3), the bias tended to be more prominent with higher percentages of involvement, which are most specific to critical patients.

Next, we studied the radiologists’ bias tendency (i.e., mean difference of overestimation) with respect to their seniority (years of experience in radiology). Correlation analysis showed a moderate Pearson coefficient of − 0.405 (*P* = 0.009) between the two, hinting that the overestimation bias decreases with experience. Simple linear regression approximations of the direction of the negative relationship are shown in Fig. 3 (*P* < 0.001). The values stay well above zero even with higher seniority, which indicates the bias spans all levels of seniority.

### Overestimation in retrospective analysis

Next, the bias was further studied in a retrospective analysis of the CT studies of 109 patients with RT–PCR-confirmed COVID-19 from HOSP-TM and EXMED. As part of the standard clinical practice, all radiological reports mentioned the total percentage of COVID-19 lung involvement.

**Figure 4:**
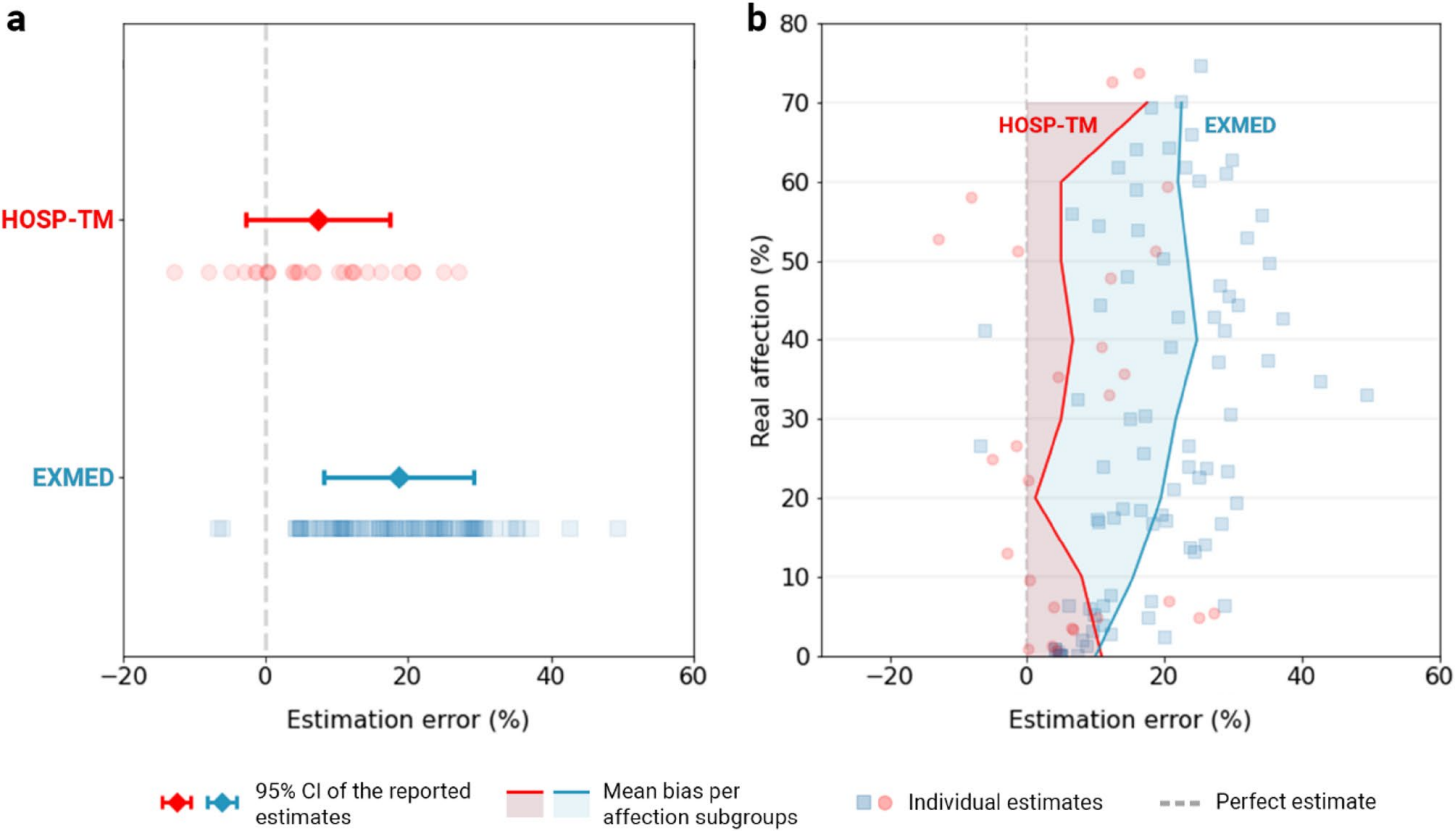
Retrospective overestimation analysis. (**a**) Mean estimation differences in CT studies from Pius Brinzeu County Emergency Hospital (HOSP-TM) and ExMed Medical Network (EXMED). (**b**) Bias disposition across severity subgroups.

Similar to the previous experiment, a visible trend emerged in both hospitals (Fig. 4). Radiologists overestimated lung involvement by 15.829 ± 6.643% (95% CI), on average. Reports from HOSP-TM exhibited a smaller bias (7.338 ± 6.227%, 95% CI) than those from EXMED (18.625 ± 6.367%, 95% CI), although both overestimations were statistically significant. The further study of large interhospital discrepancies is essential in understanding the causes and potential solutions to this bias. Therefore, to study the role of CAD in reducing this perception bias, a prospective randomised clinical trial was further conducted.

### CAD effectiveness in the clinical trial

To study the effect of CAD on the involvement assessment problem, we used the AI-PROBE protocol with *P* = 50%. AI-PROBE randomly blinded the radiologists turning AI assistance off 50% of the time. The AI analysis consisted of an automatic suggestion of total involvement percentage in addition to coloured segmentation overlays (Extended Data Figure 1). The segmentations helped the user visually check the validity of the suggested percentage and allowed for easier mental adjustments where needed.

**Figure 5:**
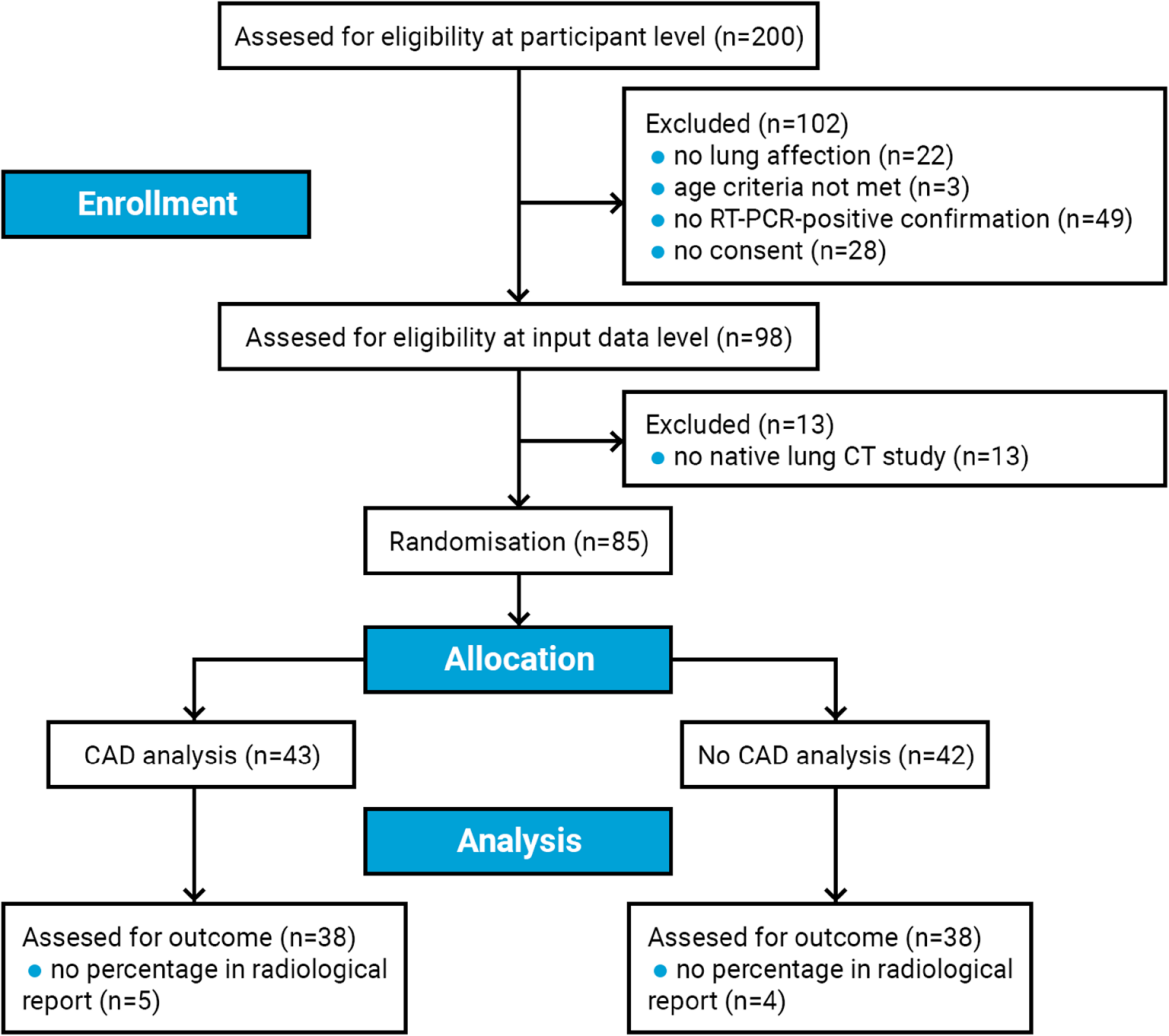
CONSORT-AI flow diagram describing the patient selection process.

A total of 85 enrolled patients were randomised between the control arm (AI intervention off) and the experimental arm (access to AI results). An additional analysis exclusion criterion eliminated 9 studies that failed to mention the involvement quantification marker from the final radiological report. Across all randomised patients, 76 CT studies (CAD access, n = 38; No CAD access, n = 38) were successfully analysed for the study outcome. This was a representative, consecutive sample of COVID-19 patients examined at HOSP-TM between 21 February 2022 and 15 March 2022 who met the inclusion criteria. The participants (37 females and 39 males) were aged 20–89 years (median = 72 years, interquartile range (IQR) = 66–81 years) and covered a wide spectrum of clinical conditions ranging from asymptomatic or milder to critical COVID-19 cases, in both inpatient and outpatient care. The results of the patient selection process are presented in Fig. 5. The study was carried out over the entire radiology department, indiscriminately of physicians’ experience level.

The patients’ true lung involvement ratios (reference standards) fit an exponential distribution (λ = 0.032). The overall mean pulmonary involvement was 32.319% (CAD access, m = 31.381%; No CAD access, m = 33.157%), and the distribution tail ended at a maximum involvement percentage of 78.030% (CAD access, M = 70.748%; No CAD access, M = 78.030%). The number of patients with other reported clinically important findings that could influence the quantitative analysis (e.g., pneumothorax, fibrosis) was also evenly distributed, with nine (23.7%) in the control group and seven (18.9%) in the intervention group. The root mean square error of the AI outputs was 4.206, with no apparent skewness between severity subgroups, as shown in Extended Data Figure 6.

**Figure 6:**
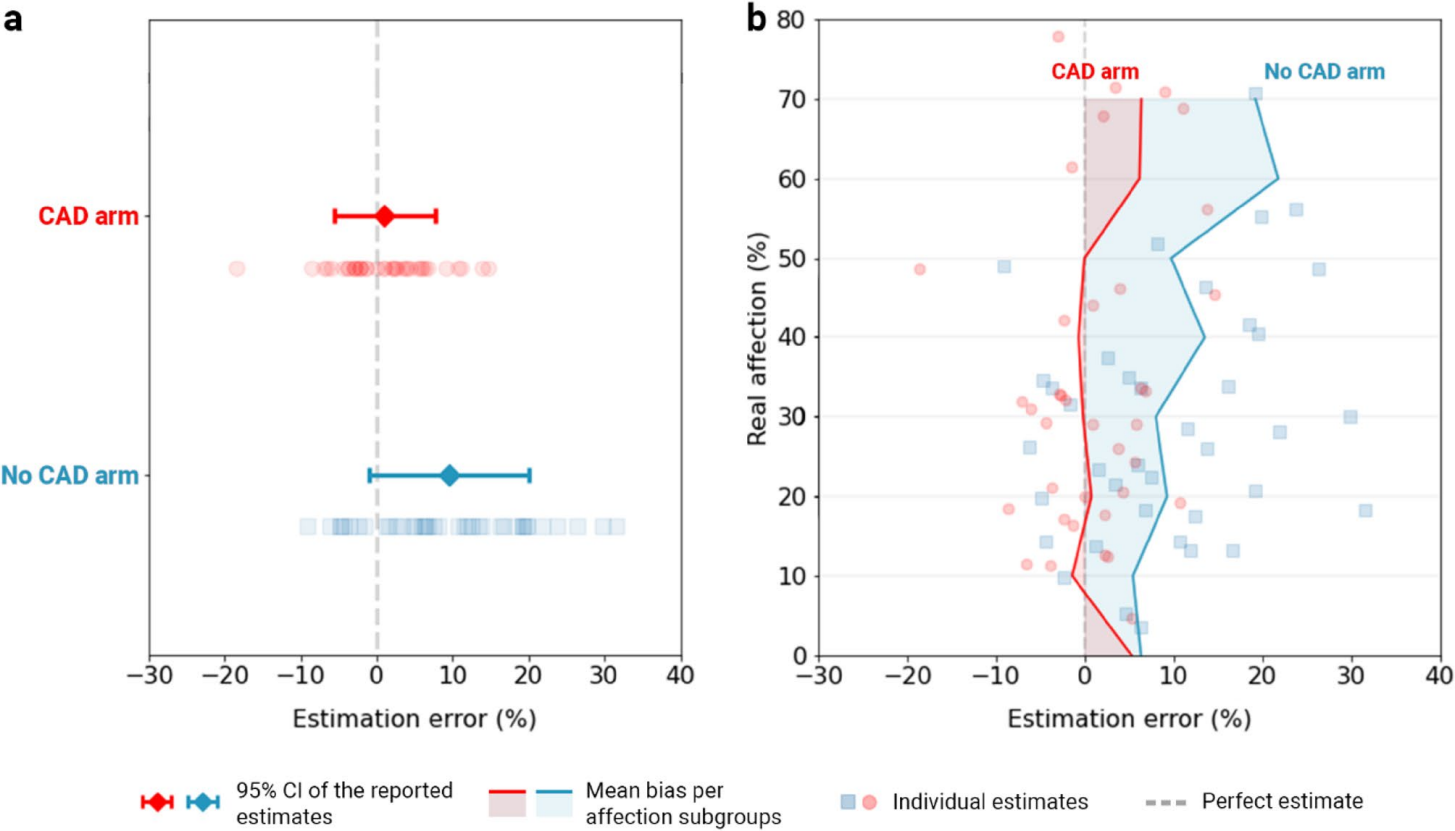
Measured estimation differences between the two arms. (**a**) Mean overestimation differences between the two arms. (**b**) Bias disposition across severity subgroups.

AI intervention reduced the mean overestimation difference from 9.471 ± 6.561 (95% CI) in the control arm, to 0.983% ± 5.181 (95% CI) (Fig. 6a). A two-tailed, two-sample t test confirmed that the difference was statistically significant (*t*(74) = -4.212, *P* < 0.001), thus rejecting H2’s null hypothesis. The overestimation bias in the controls, as well as the AI effectiveness, were also visually consistent across severity subgroups (Fig. 6b), although there was not enough statistical power to demonstrate significance.

## Discussion

This study analysed the CT quantification of lung involvement in COVID-19 in three ways. The first synthetic experiment validated the translation of a theoretical model from psychophysical science to radiology, demonstrating that radiologists are susceptible to a cognitive bias that leads to overestimating the level of involvement. The second experiment retrospectively revalidated that this cognitive bias occurs with data from real COVID-19 patients and further measured the extent of the overestimation. The results showed that the effect was even stronger in real investigations. Finally, a randomised clinical trial demonstrated that AI is a useful tool for reducing the area perception bias among radiologists.

The impact of the observed overestimation errors in the last two experiments (15.829% ± 6.643 and 9.471% ± 6.561, respectively) is substantial in a clinical context. According to previous longitudinal studies^2,17^, the mean differences between clinical severity subtypes (i.e., mild, moderate, severe, critical) were 15–25% lung involvement, on average. The bias, therefore, could become consequential when CT is used to assess clinical severity or to follow up treatment progress and misses the real involvement by 10–20%. A similar range applied to different measurements of intrapatient follow-up examinations. Nonetheless, the offset does not appear to be constant, as it depends on factors such as the clinician’s seniority, institutional type, or CAD usage, making the standardisation of treatment that much more difficult. On top of this, the bias also being present on synthetic studies and the better estimates given when the delineated maps are available, all suggest that a potential deliberate overestimation^18^ towards the safety side can be excluded as the main cause.

The bias magnitude varied considerably across the three experiments and between the two sites. A bias jump was expected when switching from the 2D data of the first experiment to the 3D data of the following two, with multiple adjustments needing to be made by the reader. This is in line with the findings of the second experiment, although not with the clinical trial results. This might be due to the lack of patients displaying above 80% lung involvement, where the bias was most pronounced in the synthetic experiment. Moreover, the second analysis showed vast differences in perception between the two hospitals. There are various possible causes for this finding, such as interhospital protocol differences when analysing COVID-19 lesions, the different types of institutions or the fact that only HOSP-TM benefited from CAD analysis software. Consequently, clinical trial showed that the use of AI might be an influential factor, as it significantly and consistently reduced the perception errors.

Lung involvement is an empirically developed measure that was previously shown to be not only predictive of patient outcomes but also decisive in establishing the patient treatment course. The study of interobserver agreement of COVID-19 scoring systems has led to varying, often positive results^19–21^ pushing the adoption further, although interobserver consistency is not indicative of real measurement validity, therefore not clinically sufficient. Moreover, research to qualify it as a valid surrogate clinical endpoint remains ongoing, as a biomarker, by definition, should be objectively measurable. Although involvement of the lungs in COVID-19 on CT is theoretically perfectly quantifiable on a pixel-to-pixel basis, this work illustrates that practical considerations make it subjective and bias-prone.

The success of the AI arm in the clinical trial is accredited in part to the widespread adoption of the CAD system within HOSP-TM. The analysis was integrated into the hospital’s picture archiving and communication system (PACS). which allowed clinicians to use AI with minimal workflow adjustment. However, not every radiologist chose to use this assistance. The AI decision support was predominantly popular among the younger radiologists, who were also the ones that demonstrated the greatest bias susceptibility. However, the exact engagement of the radiologists could not be quantitatively followed, by design. This is important, to reduce any interference and thus influence on how the CAD is perceived and used throughout the radiology department. The pragmatic design of AI-PROBE allowed studying the effect even with partial engagement of the radiology department, similar to realistic expectations inside a hospital. The interarm difference might be even more pronounced with wider adoption, although the study strived to preserve the natural adoption extent of the software.

The AI-powered CAD system was effective in mitigating perception errors. However, caution must be taken in accidentally trading the area perception bias for other AI-induced biases. AI inconsistencies in underserved patient populations are a known issue^22^. Automation bias^23,24^ is another related pitfall that roots in the overreliance of the radiologists in a computerized system. Regardless of this potential risk, the trial’s main conclusions remain unaltered, by design, as the endpoint is objectively measured on the final reports, directly. Furthermore, this study used the same AI model version for the duration of the entire trial. Inconsistencies between different versions and the effects they have on the intrahospital variability should also be studied.

Our study demonstrated that quantification of the involvement of the lungs in COVID-19 on CT scans is a perception-sensitive process prone to cognitive overestimation bias. This is of key importance given the wide use of the marker, although it was shown to be controllable with an AI decision support system. This reinforces the benefits of human-AI synergy and strengthens the need to further study the adaptability of radiology to rapid technological and methodological changes.

## Methods and materials

All procedures were conducted in conformity with the Declaration of Helsinki and International Conference on Harmonisation Good Clinical Practice guidelines. The clinical trial received approval from the Ethical Committee for Scientific Research of Pius Brinzeu County Emergency Hospital (no. 282/01/02/2022). The informed consent was collected accordingly. The retrospective analysis of data originating from EXMED received exception from informed consent (no. 14/12/02/2022) from the same committee. The clinical trial was registered on 16/03/2022 (ClinicalTrials.gov number NCT05282056).

### Preliminary analysis

To test H1 and facilitate power calculation before conducting a full prospective clinical trial, two preliminary experiments were carried out.

The first experiment involved 40 voluntary diagnostic radiologists. A call for volunteers reached physicians from eleven Romanian medical institutions. The eligibility criteria consisted of practising diagnostic radiologists of any level of experience on thoracic CTs. The participants estimated the total percentage of pulmonary deterioration in simulated CT slices based on a predelineated involvement contour (Fig. 2). This allowed only the ratio estimation step to be analysed, thus eliminating any disagreement in determining the affected areas. The slices were generated starting from an initial 90% involvement red-coloured overlay, then programmatically corroded 10% at a time using OpenCV 4.5.5^25^. The process was repeated so that a batch of nine easy/intuitive cases was matched to another nine bias-susceptible cases, which were reviewed one slice at a time in random order.

For the second experiment, a random sample of 109 studies of patients with RT–PCR-confirmed COVID-19 from HOSP-TM and EXMED were analysed retrospectively. Each study contained at least one noncontrast pulmonary CT investigation, and its corresponding radiological report, acquired between August 2021 and January 2022. The lung involvement percentages were automatically extracted using regular expressions and manually reviewed to correct for any parsing mistakes.

### Clinical trial design

To test H2, we used the AI-PROBE-2 protocol with *P* = 50%. AI-PROBE^26^ was designed to model a prospective randomised controlled clinical trial design for AI in radiology. The model randomly blinded the observers (radiologists) 50% of the time, turning AI assistance off, and showing a disclaimer instead. Therefore, the radiologists were aware of the assigned arm for every patient. Additionally, the physicians were blinded with respect to the study endpoints.

The clinical trial took place at HOSP-TM, between 21 February 2022 and 15 March 2022., where physicians were already using Rayscape, a commercial CAD system for COVID-19 volumetric quantification. The Rayscape CAD system is an existing medical device that complies with the European Economic Area regulations, it adheres to the quality management standard ISO 13485 and has obtained the CE mark. For the entire period of the study, Rayscape version v2-1.286-1.415-2.262, launched in January 2022, was used, which showed the AI analysis in the form of coloured delineated volumes along with a percentage of total lung volume deterioration, similar to standard clinical practice at the hospital. The AI analysed all CT studies in real-time and sent the analysis to the PACS. Half of the studies received the AI analysis, and the other half received the disclaimer. Aside from the disclaimer message of lacking the AI assistance in the controls during the development of the study, the radiologists did not receive any other instructions.The allocation process was performed programmatically, in real-time, by Rayscape’s Dicom Server using the default pseudorandom number generator of Python 3.8^27^.

### Data collection

The enrolment inclusion criteria included an age of 16 or older (as per the Rayscape technical requirements), a non-contrast CT examination and positive RT-PCR results confirming COVID-19. The entire enrolment flow is illustrated in Fig. 5.

Chest CT investigations were performed using NeuViz 16 Essence (Neusoft Medical Systems), Revolution EVO (GE Healthcare) and MX16 (Philips Healthcare) scanners with slice thicknesses ranging from 1.25 to 1.5 mm.

As the reference standard involvement percentages in the retrospective analysis and the clinical trial, two non-participating radiologists with at least seven years of experience in thoracic diagnostic radiology manually annotated all images at the pixel level using the ePAD^28^ platform. This bypasses the area estimation cognitive step, responsible for area judgment comparison problems and allowed for the real involvement percentages to be calculated, in turn setting the gold standard. The annotations covered GGOs, crazy paving, airspace consolidations, subpleural bands and reticular/trabecular patterns, all suggestive of COVID-19 lesions^29^. Other associated findings, such as pleural effusions, pulmonary nodules/masses, tree-in-bud patterns, tuberculosis, and pulmonary oedema (i.e., perihilar distributed GGOs or airspace consolidation) were not included.

### Statistical analysis

Based on the two preliminary experiments, we calculated that a sample size of 32 CT studies for each arm would be sufficient to detect a mean difference of 5% (alpha = 0.05, beta = 0.8) with regard to H2. To account for the risk of post-analysis exclusions, 20 extra patients were planned to be enrolled. We did not assume that the AI intervention would be noninferior in any setting; thus, two-tailed tests were used.

A two-sided paired t test was used to analyse the mean differences between the two types of samples analysed by the radiologists in the first experiment. A two-sided two-sample t test was used to analyse the bias differences between the two arms of the trial. All differences were assessed for normality both visually (Q-Q plot) and numerically (Shapiro–Wilk test).

Pearson correlation coefficient and significance value were calculated to validate the simple linear regression fitting. Despite the large residuals, the even spread of the outliers did not violate either the homoscedasticity or the normality assumptions of the regression analysis.

Data management and analysis were conducted using SciPy 1.7.3^30^ and Python 3.8.

## Supplementary Information

Supplementary Information.

## Data Availability

The raw data (CT studies, radiological reports, patient characteristics) are not publicly available, as consented by the ERBs and patients for research use only by the investigators of this study. If other authors are interested in additional experiments on the collected data, a request can be made to the corresponding author (B.A.B.) for the analyses to be made in collaboration with the current authors.

## Code availability

The Rayscape AI system is a publicly available medical device, available as a commercial software product and it’s also offered upon request for scientific enquiries. The code that generates the samples used in the first experiment (Fig. 2) could be accessed at https://github.com/RayscapeAI/Lung-CT---area-cognitive-bias-test. The CAD system’s technical design and development process does not make the subject of the study.
