# Supplementary material for: Evidence of a cognitive bias in the quantification of COVID-19 with CT: an artificial intelligence randomised clinical trial
Source: Sci Rep. 2023 Mar 25;13:4887. doi: 10.1038/s41598-023-31910-3 (PMC10039355; doi:10.1038/s41598-023-31910-3)
Supplement: Supplementary file 1 — Supplementary Information. [file 41598_2023_31910_MOESM1_ESM.pdf]

Supplementary Appendix

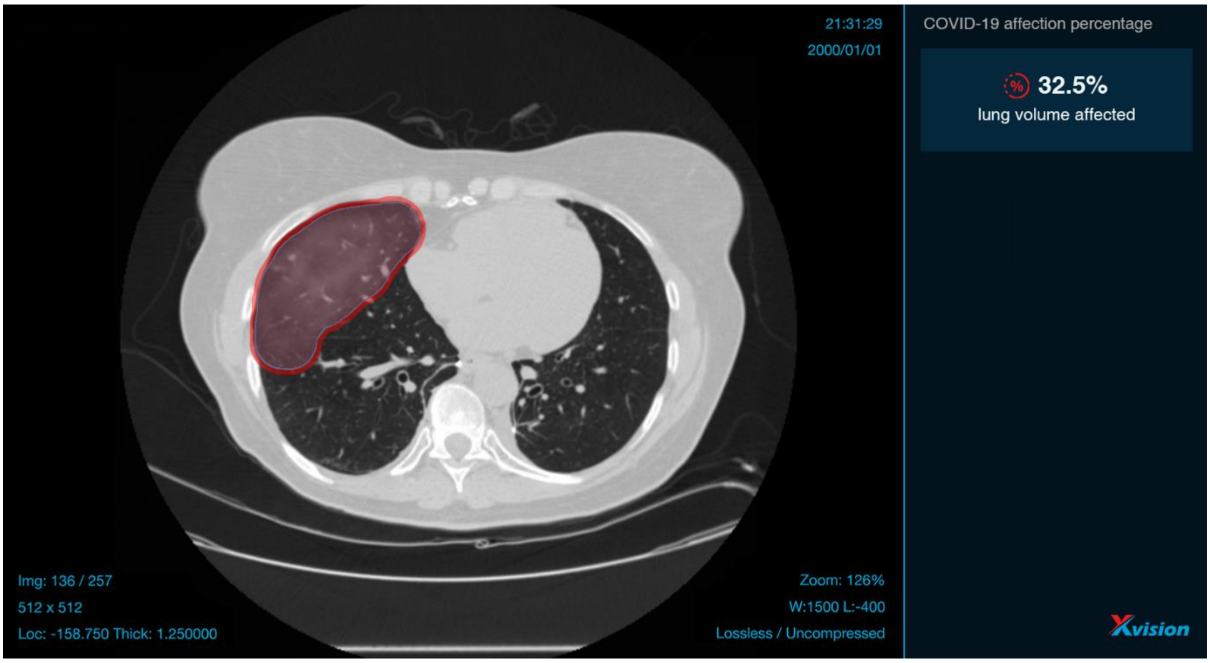

Extended Data Figure 1: *Automatically generated AI analysis report of a participating 42-year-old female patient, as integrated into the PACS. The affected area is highlighted with a red overlay on each CT slice, while the overall lung involvement percentage is displayed on the right panel.*

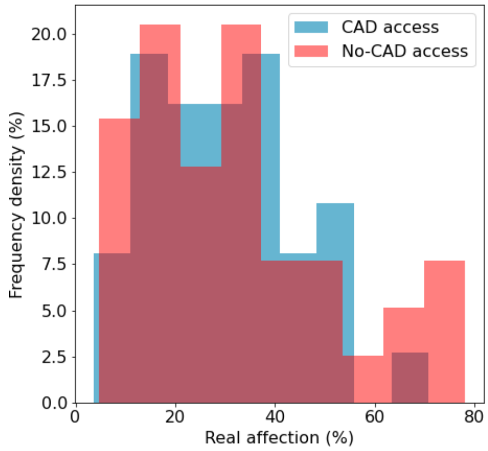

Extended Data Figure 2: *Frequency density histogram of real COVID-19 lung affection percentages over the two groups in the trial. The lower affection patients (left) are artificially under sampled because of analysis exclusion criteria (lack of reporting for minimally affected patients).*

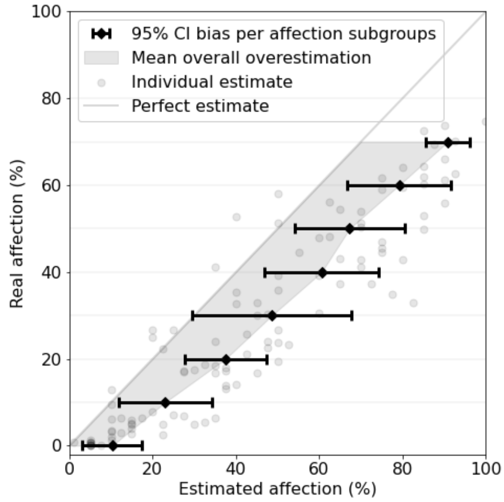

Extended Data Figure 3: *Reported overall lung involvement estimates in the second experiment, across HOSP-TM and EXMED and mean affection subgroup analysis.*

Subgroup	Patients	Estimation error (95% CI)	*P*
**Overall**	109 (100.0%)	15.83 (4.34, 27.32)	< 0.001
**Sex**			
M	51 (46.8%)	15.41 (4.58, 26.24)	< 0.001
F	58 (53.2%)	16.2 (4.07, 28.32)	< 0.001
**Age**			
25-67 yrs	55 (50.5%)	13.89 (3.36, 24.43)	< 0.001
67-93 yrs	54 (49.5%)	17.8 (5.63, 29.97)	< 0.001
**Severity**			
0-24%	55 (50.5%)	12.94 (3.64, 22.24)	< 0.001
50-74%	25 (22.9%)	17.05 (5.58, 28.51)	< 0.001
25-49%	29 (26.6%)	20.25 (6.41, 34.1)	< 0.001

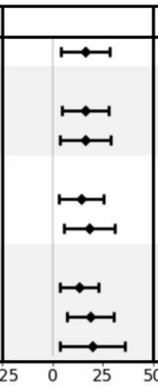

Extended Data Figure 4*: Forest plot showing subgroup analyses in the second experiment with regards to lung involvement estimation errors (difference of real and reported affection percentages). Error bars represent 95% CI; two-sided paired t-tests were performed for significance values, with Bonferroni correction.*

Subgroups	Patients CAD arm	Patients No-CAD arm	Mean errors difference (95% CI)	*P*
**Overall**	38 (50.0%)	38 (50.0%)	8.78 (4.76, 12.79)	< 0.001
**Sex**				
F	19 (25.0%)	18 (23.7%)	8.14 (2.22, 14.06)	0.064
M	19 (25.0%)	20 (26.3%)	9.29 (3.75, 14.83)	0.013
**Age**				
72-89 yrs	19 (25.0%)	18 (23.7%)	10.57 (4.93, 16.22)	0.005
20-72 yrs	19 (25.0%)	20 (26.3%)	7.15 (1.38, 12.91)	0.12
**Severity**				
25-49%	16 (21.1%)	17 (22.4%)	9.58 (2.72, 16.44)	0.061
0-24%	15 (19.7%)	17 (22.4%)	7.98 (2.48, 13.49)	0.048
50-78%	7 (9.2%)	4 (5.3%)	12.75 (4.71, 20.79)	0.075

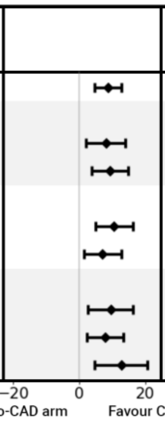

Extended Data Figure 5: *Forest plot showing subgroup analyses with regards to lung involvement estimation errors between clinical trial groups (difference of errors). Error bars represent 95% CI; two-sided, independent samples t-tests were performed for significance values, with Bonferroni correction.*

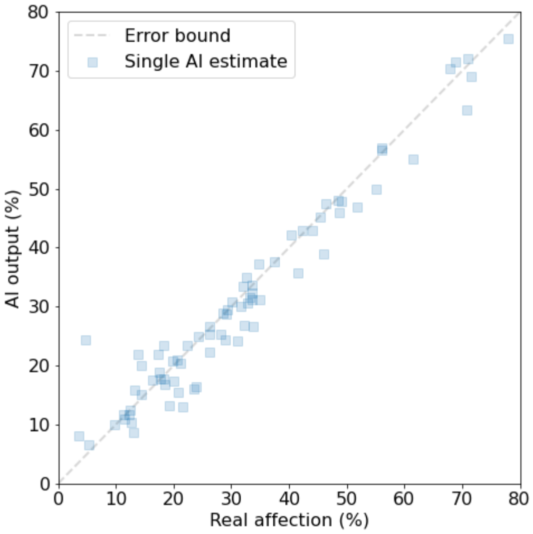

Extended Data Figure 6: *Error plot of the AI model with respect to COVID-19 lung involvement estimation.*
